# Differential Regulation of Innate and Learned Behavior by *Creb1/Crh-1* in *Caenorhabditis elegans*

**DOI:** 10.1523/JNEUROSCI.0006-19.2019

**Published:** 2019-10-02

**Authors:** Yogesh Dahiya, Saloni Rose, Shruti Thapliyal, Shivam Bhardwaj, Maruthi Prasad, Kavita Babu

**Affiliations:** Department of Biological Sciences, Indian Institute of Science Education and Research (IISER) Mohali, Knowledge City, Sector-81, S.A.S. Nagar-140306, Punjab, India

**Keywords:** *C. elegans*, chemotaxis, CREB1/CRH-1, learning, reversals

## Abstract

Memory formation is crucial for the survival of animals. Here, we study the effect of different *crh-1* [*Caenorhabditis elegans* homolog of mammalian cAMP response element binding protein 1 (CREB1)] isoforms on the ability of *C. elegans* to form long-term memory (LTM). Null mutants in *creb1/crh-1* are defective in LTM formation across phyla. We show that a specific isoform of CREB1/CRH-1, CRH-1e, is primarily responsible for memory related functions of the transcription factor in *C. elegans*. Silencing of CRH-1e-expressing neurons during training for LTM formation abolishes the LTM of the animal. Further, CRH-1e expression in RIM neurons is sufficient to rescue LTM defects of *creb1/crh-1-null* mutants. We go on to show that apart from being LTM defective, *creb1/crh-1-null* animals show defects in innate chemotaxis behavior. We further characterize the amino acids K247 and K266 as responsible for the LTM related functions of CREB1/CRH-1 while being dispensable for its innate chemotaxis behavior. These findings provide insight into the spatial and temporal workings of a crucial transcription factor that can be further exploited to find CREB1 targets involved in the process of memory formation.

**SIGNIFICANCE STATEMENT** This study elucidates the role of a specific isoform of CREB1/CRH-1, CRH-1e, in *Caenorhabditis elegans* memory formation and chemosensation. Removal of this single isoform of *creb1/crh-1* shows defects in long-term memory formation in the animal and expression of CREB1/CRH-1e in a single pair of neurons is sufficient to rescue the memory defects seen in the mutant animals. We further show that two specific amino acids of CRH-1 are required for the process of memory formation in the animal.

## Introduction

Innate behaviors are important to animals and many of these behaviors can be modified through experience. Innate behaviors, in part, represent the hardwired neural circuitry put in place during the development of an animal (Tierney, 1986; Bateson and Mameli, 2007). Mutations that affect the innate behavior could also alter the ability of animals to modify that particular behavior in response to experience. Hence, learning deficits that are attributed to certain alleles could sometimes arise because of underlying innate behavioral defects. Here, we show the effect of different *crh-1* (homologs of mammalian CREB1) mutations on innate and learned chemotaxis behaviors in *Caenorhabditis elegans*.

*C. elegans* are compost worms living on microbes as their food source. Their nervous system consists of 302 neurons. They show a variety of robust and adaptable behaviors like chemotaxis and thermotaxis (for review, see Hart, 2006; Ardiel and Rankin, 2010). *C. elegans* move in a sinusoidal wave pattern. Under laboratory conditions, their continuous forward movement is punctuated by frequent stops and events of backward movement called reversals. After each reversal, the probability of change in direction of movement increases significantly. One of the strategies deployed by *C. elegans* to move in response to a chemical gradient is to change the frequency of reversals in response to a gradient (Pierce-Shimomura et al., 1999).

CREB1 (cAMP response element binding protein 1) was initially characterized for its role in long-term facilitation of the gill withdrawal reflex in *Aplysia* (Dash et al., 1990). Neural activity-dependent CREB1 activation in a defined set of neurons is one of the requirements for the process of long-term memory (LTM) formation (for review, see Flavell and Greenberg, 2008). CREB1-null mutants are shown to be defective in LTM formation across phyla (for review, see Silva et al., 1998).

*C. elegans* have innate preferences for certain odors. They detect metabolic byproducts of microbes as cues for finding their food. For example, under well fed naive condition worms are attracted to AWC sensed odorants like isoamyl alcohol (for review, see Bargmann, 2006). However, this odor to behavior relationship can be modified if worms are exposed to periods of starvation along-with chemo-attractants (Pereira and van der Kooy, 2012). Likewise, neutral concentrations of butanone can be turned attractive when repetitively paired with food. These modified behaviors persist for 40 h after training (Kauffman et al., 2011). The role of *crh-1* in LTM formation has been described using a variety of training paradigms using the *crh-1-null* mutant strain *crh-1(tz2*) (Kauffman et al., 2010; Nishida et al., 2011; Timbers and Rankin, 2011; Lau et al., 2013; Jin et al., 2016). Chemotaxis behavior is generally used to assess the performance of memory formation and retrieval assays. Chemotaxis in *C. elegans* is dependent on a large number of subtle behaviors like reversals and head bends (Pierce-Shimomura et al., 1999; Iino and Yoshida, 2009; Larsch et al., 2015). We have observed that the innate ability of worms to modulate reversals in response to external stimuli is a critical factor in determining the performance of *C. elegans* in chemotaxis assays, thereby affecting the readout of learning assays.

The role of *crh-1* in defining LTM in *C. elegans* has only been partially understood. First, given its pleiotropy of function, it is unclear which *crh-1* isoforms are involved in memory related functions. Second, the motifs on CRH-1 protein specifically involved in memory related processes are unknown. Third, the identity of neurons where *crh-1* is required for memory formation is largely unknown. Here, we used CRISPR-based mutagenesis to dissect out memory related functions of CREB1/CRH-1. Our experiments show that two of the six potential isoforms of CRH1, CRH-1c, and CRH-1e, are responsible for these functions. Further, amino acid residues Lys247 and Lys266 are critical for memory related functions of CRH-1 while being dispensable for innate chemotaxis behavior. We have also identified RIM and/or AVE as the minimal set of neurons required for memory related functions of CRH-1.

## Materials and Methods

### 

#### Strains

*C. elegans* were maintained using standard methods (Brenner, 1974). Wild-type N2 and *crh-1(tz2*) mutant lines were obtained from the *Caenorhabditis* Genetics Center (University of Minnesota, Minneapolis, MN). The primers, plasmids and strains used in this study are listed in Tables 1, 2, and 3, respectively.

**Table 1.** List of primers used in this study

Primer ID	Sequence	Primer type	Gene
qYD21	GCCACTTCGCCGTTGATGATG	qPCR Forward	*crh-1a*
qYD20	TCCTCCGGCTCCTTCTTCATC	qPCR Reverse	*crh-1a*
qYD179	GTAGATGCTTCACCATTACAGTTT	qPCR Forward	*crh-1b*
qYD23	GGCAAGACGAGTGGCTGATT	qPCR Reverse	*crh-1b*
qYD22	ATGTCAGCGAAAGGTAACGGATC	qPCR Forward	*crh-1c*
qYD20	TCCTCCGGCTCCTTCTTCATC	qPCR Reverse	*crh-1c*
qYD24	GTACCCAACAGCAACACGG	qPCR Forward	*crh-1d*
qYD25	CCGCCGTTTCTAGATCTTTGAG	qPCR Reverse	*crh-1d*
qYD19	ATGATGTTCCTCAGGGCATTACAA	qPCR Forward	*crh-1e*
qYD20	TCCTCCGGCTCCTTCTTCATC	qPCR Reverse	*crh-1e*
qYD26	ATGGAGTCACTGGTTTTCAATGG	qPCR Forward	*crh-1f*
qYD27	GTACGGATTGTTGTTGGGATGG	qPCR Reverse	*crh-1f*
qYD09	TACTCTTTCACCACCACCGC	qPCR Forward	*act-1*
qYD10	ACGGTGATGACTTGTCCGTC	qPCR Reverse	*act-1*
YD157	TGGAAGGAGGAGGAGATGGAAA	Genotyping Forward (external)	*crh-1(tz2)*
YD158	GCAGTACAGCTCTTTCAGCGTT	Genotyping Forward (internal)	*crh-1(tz2)*
YD159	AATTCGGCACAACGGACTGG	Genotyping Reverse (external)	*crh-1(tz2)*
YD172	ATTACCTGCAGGCTGCAGGTCGACTCTAGAG	Cloning Forward	p*hsp16.41*
YD173	GGCAGCTAGCATTTTTTCTACCGGTACCAATAC	Cloning Reverse	p*hsp16.41*
YD165	ATTAGCTAGCATGGCCACAATGGCGAGCAC	Cloning Forward	*crh-1a*
YD164	ATTACCATGGTCACATTCCGTCCTTTTCCTTTC	Cloning Reverse	*crh-1a*
YD165	ATTAGCTAGCATGGCCACAATGGCGAGCAC	Cloning Forward	*crh-1b*
YD164	ATTACCATGGTCACATTCCGTCCTTTTCCTTTC	Cloning Reverse	*crh-1b*
YD166	ATTAGCTAGCATGTCAGCGAAAGGTAACGGAT	Cloning Forward	*crh-1c*
YD164	ATTACCATGGTCACATTCCGTCCTTTTCCTTTC	Cloning Reverse	*crh-1c*
YD167	ATTAGCTAGCATGGCCACAATGGCGAGCAC	Cloning Forward	*crh-1d*
YD164	ATTACCATGGTCACATTCCGTCCTTTTCCTTTC	Cloning Reverse	*crh-1d*
YD168	ATTAGCTAGCATGATGTTCCTCAGGGCATTACA	Cloning Forward	*crh-1e*
YD164	ATTACCATGGTCACATTCCGTCCTTTTCCTTTC	Cloning Reverse	*crh-1e*
YD169	ATTAGCTAGCATGGAGTCACTGGTTTTCAATGG	Cloning Forward	*crh-1f*
YD164	ATTACCATGGTCACATTCCGTCCTTTTCCTTTC	Cloning Reverse	*crh-1f*
YD180	CATAATAACCGGTAAAATGTACCCATACGACGTTCCAG	Cloning Forward	3xHA
YD181	CATAGCTAGCAGCGTAATCTGGGACGTCA	Cloning Reverse	3xHA
YD320	ATCAGCATGCATCTTCAGATGGGAGCAGTGG	Cloning Forward	p*rab-3*
YD321	GCATGGATCCAAACTTGTCATCTGAAAATAGGGCT	Cloning Reverse	p*rab-3*
YD260	ATAGCCTGCAGGAAGTGGACACTGAGAGAGAGAG	Cloning Forward	p*nmr-1*
YD261	ATAGCCCGGGCTGTAACAAAACTAAAGTTTGTCGTGTTC	Cloning Reverse	p*nmr-1*
AB139	GCGTCGACAAGTGACACCACGCTCACA	Cloning Forward	p*rig-3*
AB140	CCCCCCGGGAGCTGTGAAATTTTTAGGCAGT	Cloning Reverse	p*rig-3*
YD313	ACGTGCATGCGTTAAATATGTGGCCACAATGACAATTAT	Cloning Forward	p*gcy-13*
YD314	ATCAGGATCCGTCCTGAAAAATTATTGAAAGTTTGTAATAAG	Cloning Reverse	p*gcy-13*
YD253	ATACCTGCAGGGAATTTAATAACTAATACATATTATTTGGCACACTC	Cloning Forward	p*crh-1e*
YD254	ATAGGATCCATTATTCAGAAATTGAATAGAGAATTAGAATTAGAAAC	Cloning Reverse	p*crh-1e*
YD318	CTGCAAGCTTCACTTTTCTACGTTTCCATAATAATTACATAG	Cloning Forward	p*crh-1a*
YD319	ATACGGATCCAGCCGTGAGATGTCCGC	Cloning Reverse	p*crh-1a*
YD205	AGTAAGCTTGTTTACGCTCGCGAGGAGAC	Cloning Forward	p*str-2*
YD206	AGTCTGCAGTTTTATGGATCACGAGTATTCG	Cloning Reverse	p*str-2*
crYD35	ATCAGGTCTCCTCTTCTTTTCTAATACTTACCCTGGTTTTAGAGCTAGAAATAGCAAG	Guide RNA	*crh-1e* deletion
crYD65	ATCAGGTCTCCTCTTCAAAATGTCAGCGAAAGGTAAGTTTTAGAGCTAGAAATAGCAAG	Guide RNA	*crh-1c* deletion
crYD48	ATCAGGTCTCCTCTTGAGCATCATCAACGGCGAAGGTTTTAGAGCTAGAAATAGCAAG	Guide RNA	*crh-1a,b,d* deletion
crYD106	ATCAGGTCTCCTCTTTGGGCTCGAGCTGTAGCCGCGTTTTAGAGCTAGAAATAGCAAG	Guide RNA	*crh1*(K247R/K266R)
crYD01	GTTTCTCGAGCCATGGTTACCGGTCTC	Guide RNA	Universal
crYD44	ATACGCATGCACACCTAAAATTTTCAGATTGAGTCTGG	Forward	5′ Homology arm *crh-1e* deletion
crYD45	ATCAACTAGTTGCGCCCTGTTGAACTGGGA	Reverse	5′ Homology arm *crh-1e* deletion
crYD46	ATCAGGGCCCAGAAAAGATTAGTGTGTGTGTGTTTG	Forward	3′ Homology arm *crh-1e* deletion
crYD47	ATCACTTAAGCGAGTTGGTTTTATTACACAGCAGAG	Reverse	3′ Homology arm *crh-1e* deletion
crYD59	ATCAACATGTGCGAATCGCTGATTGGTTGCAATTC	Forward	5′ Homology arm *crh-1c* deletion
crYD60	ATCAACTAGTTTTATACTCGTTTTGGTGTGCAAAAAGGC	Reverse	5′ Homology arm *crh-1c* deletion
crYD61	ATCAGGGCCCAATGACACAAGAATGCGAAAACATCT	Forward	3′ Homology arm *crh-1c* deletion
crYD62	ATCACTTAAGTTCGGCCAATTTTGCGAATTTTAAG	Reverse	3′ Homology arm *crh-1c* deletion
crYD50	ATCAGCATGCTCTCTTTCACCGCCAATTTTTGTG	Forward	5′ Homology arm *crh- 1a,b,d* deletion
crYD51	ATCAACTAGTGAGATGTCCGCCACTTATTTTTGTTTA	Reverse	5′ Homology arm *crh- 1a,b,d* deletion
crYD52	ATCAGGGCCCAGTAGAGTTTTAGTGGAAAAATTTCGAG	Forward	3′ Homology arm *crh- 1a,b,d* deletion
crYD53	ATCACTTAAGCGATATGAGGGCCTCCTATTAAGTT	Reverse	3′ Homology arm *crh- 1a,b,d* deletion
crYD74	ACTCGCATGCGAAGTGCAATGAAGCCAATGTTGG	Forward	5′ Homology arm *crh- 1*(K247R/K2 66R)
crYD90	ACTAACTAGTCAAATTTCCGCTCCAAA	Reverse	5′ Homology
	AAATTACCTTACGTTTTCTGCGGCACTCTTTCGCTGCCTCTCGATT		arm *crh- 1*(K247R/K2 66R)
crYD91	ACTCGGGCCCGAGGGAAAAAAATTATTTTTTGGCTGAAAAATTGA	Forward	3′ Homology arm *crh- 1*(K247R/K2 66R)
crYD92	ACGACTTAAGTGAGTGCTTTGTTCTGATTTTCCAGC	Reverse	3′ Homology arm *crh- 1*(K247R/K2 66R)

**Table 2.** List of plasmids used in this study

S. no.	Plasmid ID	Plasmid
1	pBAB713	p*rab-3*::CRH-1a
2	pBAB714	p*rab-3*::CRH-1b
3	pBAB715	p*rab-3*::CRH-1c
4	pBAB716	p*rab-3*::CRH-1d
5	pBAB717	p*rab-3*::CRH-1e
6	pBAB719	p*rab-3*::CRH-1f
7	pBAB712	p*hsp16.41*::CRH-1e
8	pBAB726	p*nmr-1*::CRH-1e
9	pBAB746	p*crh-1e*::CRH-1e
10	pBAB748	p*rig-3*::mCherry
11	pBAB749	p*opt-3*::mCherry
12	pBAB750	p*gcy-13*::mCherry
13	pBAB747	p*rig-3*::CRH-1e
14	pBAB745	p*opt-3*::CRH-1e
15	pBAB744	p*gcy-13*::CRH-1e
16	pBAB761	p*str-2*::ChR2::YFP
17	pBAB751	p*crh-1e*::GFP
18	pBAB736	p*nmr-1*::mCherry
19	pBAB752	p*crh-1a*::GFP
20	pBAB741	p*crh-1e*::HisCl1::sl2::GFP
21	pBAB709	Repair template *crh-1a,b,d* deletion
22	pBAB711	Repair template *crh-1c* deletion
23	pBAB708	Repair template *crh-1e* deletion
24	pBAB760	Repair template *crh-1*K247R/K266R point mutation
25	pBAB757	Repair template 3xHA::CRH-1e
26	pBAB758	Repair template 3xHA::CRH-1c
27	pBAB759	gRNA *crh-1a,b,d* deletion
28	pBAB762	gRNA *crh-1c* deletion
29	pBAB763	gRNA *crh-1e* deletion
30	pBAB756	gRNA *crh-*1K247R/266R point mutation
31	pBAB764	gRNA 3xHA::CRH-1c
32	pBAB765	gRNA 3xHA::CRH-1e

**Table 3.** List of strains used in this study

Strain	Genotype	Description
BAB701	*crh-1(tz2)* (CGC strain YT17)	From CGC (outcrossed 2X)
BAB713	*crh-1;* p*rab-3*::CRH-1a	Array no. IndEx713
BAB714	*crh-1;* p*rab-3*::CRH-1b	Array no. IndEx714
BAB715	*crh-1;* p*rab-3*::CRH-1c	Array no. IndEx715
BAB716	*crh-1;* p*rab-3*::CRH-1d	Array no. IndEx716
BAB717	*crh-1;* p*rab-3*::CRH-1e	Array no. IndEx717
BAB708	CRISPR based deletion corresponding to the first exon of *crh-1e* mRNA	*crh-1*(Ind708 + loxP)
BAB709	CRISPR deletion corresponding to the first exon of *crh-1a,b,d* mRNAs	*crh-1*(Ind709 + loxP)
BAB710	CRISPR based deletion corresponding to the first exon of *crh-1c* and *crh-1e* mRNAs	*crh-1*(Ind710 + loxP)
BAB711	CRISPR based deletion corresponding to the first exon of *crh-1c* mRNA	*crh-1*(Ind711 + loxP)
BAB719	p*rab-3*::CRH-1f	Integrated line IndIs719
BAB726	*crh-1;* p*nmr-1*::CRH-1e	Array no. IndEx726
BAB736	p*nmr-1*::mCherry; p*crh-1e*::GFP	Array no. IndEx736
BAB703	p*rig-3*::mCherry; p*crh-1e*::GFP	Array no. IndEx703
BAB704	*popt-3*::mCherry; p*crh-1e*::GFP	Array no. IndEx704
BAB705	*pgcy-13*::mCherry; p*crh-1e*::GFP	Array no. IndEx705
BAB747	*crh-1;* p*rig-3*::CRH-1e	Array no. IndEx747
BAB745	*crh-1;* p*opt-3*::CRH-1e	Array no. IndEx745
BAB744	*crh-1;* p*gcy-13*::CRH-1e	Array no. IndEx744
BAB746	*crh-1;* p*crh-1e*::CRH-1e	Array no. IndEx746
BAB741	p*crh-1e*::HISCl1::SL2::GFP	Array no. IndEx741
BAB712	*crh-1;* p*hsp16.41*::CRH-1e	Array no. IndEx712
BAB754	CRISPR based addition of 3xHA::CRH-1c and e	*crh-1*(Ind754 [3xHA::*crh-1c*+loxP; 3xHA::*crh-1e*+loxP])
BAB760	CRISPR based substitution of K247R/266R	*crh-1*(Ind760 [K247R/K266R]+loxP)
BAB735	p*nmr-1*::mCherry; p*crh-1a*::GFP	Array no. IndEx735
BAB761	p*str-2*::ChR2::YFP; p*rig-3*::GCaMP5G	Array no. IndEx761
BAB762	BAB760; p*str-2*::ChR2::YFP; p*rig-3*::GCaMP5G	*crh-1*(Ind760 [K247R/K266R]+loxP) with array

#### Constructs and transgenes

The *crh-1* isoforms (*crh-1a-f*) were cloned into the pPD49.26 vector backbone. *crh-1b* and *crh-1c* cDNAs were synthesized from Sigma-Aldrich and the rest were obtained by reverse transcription and PCR using wild-type (WT) *C. elegans* RNA. N2 (WT) or *crh-1(tz2*) mutant lines were used for transforming the CRH-1 isoforms. Transformations were done as described previously (Mello et al., 1991). The rescue constructs and the promoter fusion constructs were injected in concentrations of 10–20 ng/μl. p*myo-2*::mCherry (2 ng/μl) or p*vha-6*::mCherry (10 ng/μl) or p*unc-122::*GFP (25 ng/μl) were used as coinjection markers. mCherry and GFP cDNA were cloned from pCFJ90 and pPD95.75, respectively. The primers used for the different cloning experiments are tabulated in Table 1.

#### Behavioral assays

##### Conditioning with IAA/diacetyl and high temperature.

**Figure 1.**
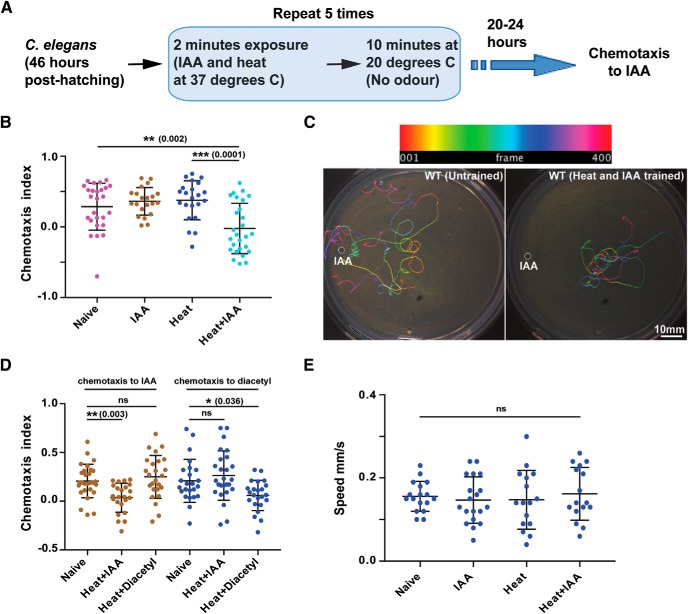
Associative learning in *C. elegans*. ***A***, Schematic flowchart of the *C. elegans* training routine followed in this study. ***B***, Scatter plot showing the effect of IAA and heat on WT *C. elegans*. ***C***, Representative images of typical chemotaxis tracks. Top, Color-coding of tracks as a function of frame number. ***D***, Scatter plot indicating the specificity of learning. Animals were trained with Heat+IAA or Heat+Diacetyl and their chemotaxis indices were plotted. ***E***, Plot showing speed of worm movement when subjected to different training conditions. Error bars are SD. Multiple comparisons were done using one-way ANOVA and *p* values were adjusted using Dunnett's correction method. In graphs of all figures significant *p* values are added in brackets next to the significance asterisks (*) and “ns” indicates not significant. See Figure 1-1.

A dry heating block was maintained at 37°C. Twenty microliters of 10% isoamyl alcohol (IAA) or 1% diacetyl (both diluted in ethanol) was kept on a piece of cover glass on the heating block. A Petri-plate containing *C. elegans* on OP50 lawn was inverted onto the setup for 2 min. The training cycle was repeated five times with an inter-training interval of 10 min (illustrated in Fig. 1*A*).

##### Chemotaxis assay and behavioral analysis.

Trained animals were kept at 20°C for 20–24 h unless mentioned otherwise. *C. elegans* were picked using an eyelash pick and allowed to crawl on an unseeded nematode growth medium (NGM) plates for 30 s. Four to six animals were gently transferred to the center of 90 mm NGM agar plates (without food). One microliter of odorant [IAA (1%)/diacetyl (0.1%)/benzaldehyde (1%)] was kept at one end of the plate. Chemotaxis behavior was recorded for 10 min using a 5 megapixel CMOS USB camera (Mightex) at 2 frames/s using the Mightex Camera Demo v1.1.0 software. Recordings were done in a Peltier cooled incubator at 20° Celsius. Videos were analyzed using FIJI software (Schindelin et al., 2012). To quantify their chemotaxis behavior, a non-dimensional index based on individual *C. elegans* trajectory was used (Eq. 1). For the attractant, displacement was positive if the animal traveled up the gradient while it was negative if the animal traveled down the gradient (Luo et al., 2014). To quantify the reversal behavior of *C. elegans*, the worms were allowed to crawl in presence or absence of an IAA gradient for 10 min. Videos were recorded at 2 frames/s. Reversals were calculated manually by analyzing the videos.

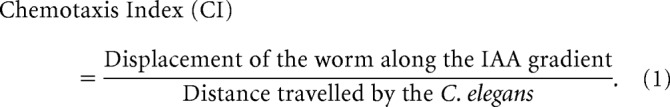


#### Quantitative PCR

Total RNA was isolated using Trizol from WT animals grown to the young adult stage. Fifty nanograms of total RNA was used for cDNA synthesis. qPCR was performed using a Qiagen SYBR real time PCR kit. Isoform-specific primers were used for amplification of the different *crh-1* isoforms. The primers used are listed in in Table 1. qPCR was done using the Roche Light Cycler 480. *C_t_* values were calculated using Equation 2 with the Roche software. The data were analyzed using Δ*C* method (Eq. 3).

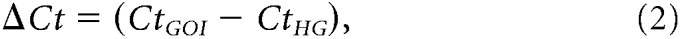


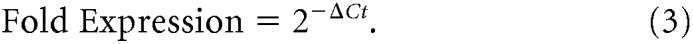
 GOI indicates gene of interest, and HG indicates the housekeeping gene, *act-1*.

#### Fluorescence microscopy

Young adult hermaphrodites were used for imaging. Animals were mounted on 2% agarose pads with 10 mm sodium azide solution in M9 medium. Images were acquired on a Leica SP6 upright laser scanning confocal microscope using the 40× oil-immersion objective lens. All images were processed and analyzed by Fiji.

#### Histamine supplementation

Histamine-dihydrochloride (1 m; Sigma-Aldrich, catalog #53300) filter sterilized stock solution was prepared in distilled water. Final working concentrations (10 mm) were used for all experiments. Histamine-supplemented NGM plates were prepared as described previously (Pokala et al., 2014).

#### Optogenetics

The *str-2* promoter was used to drive AWC neuron-specific expression of channelrhodopsin-2 (ChR2). Animals expressing ChR2 were grown on NGM agar plates seeded with OP50-containing 100 μm all-trans retinal. The *C. elegans* were grown in the dark until late L4/early adult stages. The assay was performed on freshly seeded NGM plates. During the assay, ChR2 was excited by blue light (460–490 nm) using an epifluorescence unit (U-HGLGPS, Olympus) attached to the Nikon SMZ2000 microscope. Blue light was illuminated for 3 s and reversal events were quantified. If any worm executed reverse movement for one body length, it was counted as a reversal event. Each worm was illuminated with blue light 8 times with 20 s intervals between subsequent stimulation. Reversal probability was calculated by dividing the number of reversal events with number of optogenetic stimulations. Each dot in the scatter plot indicates the reversal probability of a single *C. elegans* under observation. The results were plotted as mean SD and evaluated using the standard Student's *t* test. The experimenter was blind to the genotypes of the strains while performing these experiments.

#### Calcium imaging

The genetically encoded Ca^+2^ indicator GCaMP-expressing strain p*rig-3*::GCaMP5 was used to visualize Ca^+2^ transients in the AVA command interneuron (Larsch et al., 2013). A 0.2 μl drop of polystyrene beads (0.1 μm) was added on the top of 10% agarose pad (prepared in M9). A single worm was kept on the drop containing beads. The animal was immobilized by putting a circular cover glass over it. ChR2 was excited by blue light (460–490 nm) using an epifluorescence unit (U-HGLGPS, Olympus) and simultaneously calcium transients were recorded for 3.0 s using Micro-Manager software at a speed of 10 frames/s (Edelstein et al., 2014). Image analysis was done using Fiji. A ROI was drawn over the AVA cell body. Fluorescence (*F*) was calculated by subtracting the background fluorescence (*F*_bkgd_) value from the mean fluorescence (*F*_mean_) of ROI. The fluorescence value was estimated for each frame by manual repositioning of the ROI. Calcium transients were plotted as *F*/*F*_o_, where *F* is the change in the fluorescence value (*F*) from its baseline fluorescence (*F*_o_). Fluorescence intensity of second frame (*t* = 200 ms) was taken as baseline fluorescence (*F*_o_).

#### CRISPR-based genome editing

CRISPR was used to create *crh-1* isoform mutations as described previously (Dickinson and Goldstein, 2016). Briefly, the selection excision cassette (SEC) from the plasmid pDD287 was cloned along-with flanking loxP sites into pPD95.75. The resulting plasmid was used to clone homology arms (500–1500 bp) and the desired genetic modification using restriction enzyme-based cloning methods. A 20 bp guide RNA was cloned into pRB1017. The plasmid mixture containing repair template (50 ng/μl), sgRNA (20 ng/μl), pJW1259 (50 ng/μl), pCFJ90 (2.5 ng/μl), and p*vha-6*::mCherry (10 ng/μl) was injected into 20–30 adult hermaphrodite animals (containing 4–5 eggs). The *C. elegans* were kept at 20°C. After 60 h of injection, the antibiotic hygromycin (250 μg/ml) was added directly to the plates containing the injected worms. The *C. elegans* were left at 20°C for 10 d. After 10 d, 10–15 non-fluorescent roller animals were singled out onto regular NGM plates. To remove SEC 30–40 L1–L2 animals from plates with 100% roller progeny were kept at 34°C for 3–4 h. Normal moving worms were isolated and target DNA was sequenced to analyze and confirm desired modifications.

#### Statistical analysis

All statistical analyses were done using GraphPad Prism 7. Outliers were identified using Grubbs method (α = 0.05). Mean values were compared using one-way ANOVA. Bonferroni or Dunnett correction was used to adjust *p* values for multiple comparisons. Error bars represent SD except for Figure 5*E* where they represent SEM. The level of significance was set as *p* ≤ 0.05.

## Results

### Associative learning in *C. elegans* using isoamyl alcohol and high temperature

Previous studies have largely examined associative LTM formation in *C. elegans* by pairing the presence/absence of food (unconditioned stimulus) with a variety of cues (Amano and Maruyama, 2011; Kauffman et al., 2011; Nishida et al., 2011). Under these previously reported training conditions *C. elegans* could retain memory for upto 40 h (Kauffman et al., 2011). Because studies have shown that starvation alone in the absence of any external stimulus is sufficient to induce the expression of CREB1/CRH-1 in *C. elegans* (Suo et al., 2006, 2009), we were interested in developing a training paradigm that was independent of the feeding state of the animal. To this end we went on to develop a learning paradigm using IAA and high temperature.

*C. elegans* were exposed to the vapors of IAA at high temperatures (37°C) for 2 min followed by a rest period of 10 min at 20°C, repeated five times (illustrated in Fig. 1*A*). After 20–24 h, the animals were allowed to crawl on a 90 mm NGM agar plate in response to an IAA gradient. We observed significantly reduced chemotaxis index (CI) values for *C. elegans* exposed to IAA at 37°C (Figs. 1*B*, plate images shown in *C*, and Fig. 1-1*A*,*B*), whereas the CI values for *C. elegans* exposed only to 37°C temperature or IAA alone were comparable to naive animals (Fig. 1*B*). To understand whether learning was specific to the cues provided during training, we trained worms with heat and IAA followed by chemotaxis to diacetyl after 20–24 h. We also did experiments where we used the training paradigm with heat and diacetyl and then went on to test for chemotaxis to IAA after 20–24 h. IAA when paired with heat resulted in reduced chemotaxis to IAA, whereas chemotaxis to diacetyl was unaffected and diacetyl when paired with heat resulted in reduced chemotaxis to diacetyl while chemotaxis to IAA was unaffected (Fig. 1*D*). Animals trained with heat and IAA showed normal chemotaxis in response to 1% benzaldehyde, another odorant sensed by AWC neurons, negating a generic functional downregulation of AWC neurons in response to the training paradigm (Fig. 1-1*C*). To negate the potentially harmful effects of the chemicals/high temperature used in training we measured the average crawling speed of *C. elegans* that were subjected to various training conditions, these animals crawled at speeds comparable to naive animals (Fig. 1*E*).

10.1523/JNEUROSCI.0006-19.2019.f1-1 Figure 1-1 **Chemotaxis indices in *C. elegans* after training the animals (A)** Cartoon of a typical chemotaxis plate. 4-6 *C. elegans* were kept near the center of the plate and 1 μl of 1% IAA is kept at one end of the plate (dashed circle). A video was recorded as the animals moved for 10m, using a 5 megapixel USB camera at 2 frames/s. The *C. elegans* tracks were analyzed and chemotaxis indices were calculated. **(B)** Cartoon of calculation of *C. elegans* displacement using worm tracks. The worm tracks were generated by FIJI using a temporal color code. The distance travelled by the *C. elegans* was measured by following the track using the segmented line tool in FIJI. In order to measure the displacement in the direction of the attractant, the shortest distance from the end of the track to the source of the attractant was measured and this value was subtracted from the shortest distance from the start of the track to the source of the attractant. The chemotaxis index was calculated by dividing the displacement in the direction of the attractant by the total distance travelled by the *C. elegans*. **(C)** Scatter plot testing the specificity of learning in worms. Animals were trained with Heat and IAA and were tested for chemotactic behavior in response to IAA or Benzaldehyde. Their chemotaxis indices were then plotted as shown. Error bars are SD. Multiple comparisons were done using one-way ANOVA and p-values were adjusted using Dunnetts correction method. **(D)** This panel indicates sequence alignments of CRH-1 isoforms a-f using MUSCLE. The N-terminal part of the protein having maximum sequence variation is shown. The alignment was generated using the UGENE software. Download Figure 1-1, TIF file

### LTM formation in *C. elegans* requires CRH-1c and CRH-1e

**Figure 2.**
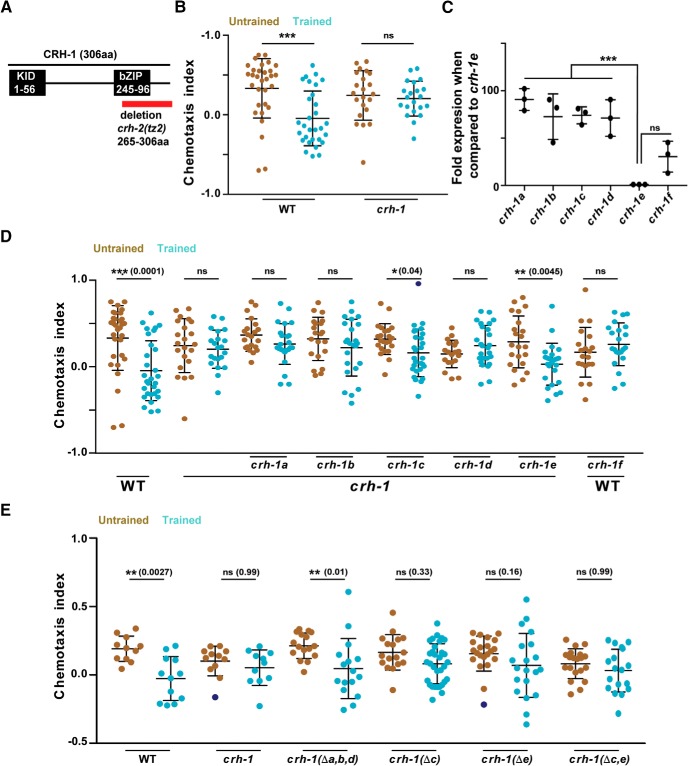
CRH-1c and e are required for associative learning in *C. elegans*. ***A***, Schematic showing *C. elegans* CRH-1 and the deletion in the mutant. Two major functional domains KID and bZIP (DNA-binding domain) domains are depicted. The red bar shows the deletion found in the *crh-1-null* mutant *tz2*. This deletion affects all CRH-1 isoforms. ***B***, Scatter plot showing chemotaxis indices of WT and *crh-1-null* mutants. ***C***, Graph showing quantitative PCR results comparing mRNA expression levels of different CRH-1 isoforms to the CRH-1e isoform in young adult WT *C. elegans*. ***D***, Scatter plot showing chemotaxis indices of WT, *crh-1-null* mutants, and pan-neuronal CRH-1 rescue lines using different *crh-1* isoforms. ***E***, Scatter plot showing chemotaxis indices of WT, *crh-1-null* mutants and *crh-1* isoform deletion lines. Error bars are SD. Multiple comparisons were done using one-way ANOVA and *p* values were adjusted using Bonferroni (***B***, ***D***, ***E***) and Dunnett's (***C***) methods. Data points represented with dark blue dots in the scatter plots represents outliers identified using Grubb's method (α = 0.05). **p < 0.01, ***p < 0.001, ns, *p* > 0.05.

The *creb1/crh-1* gene is pleotropic in function, known to be involved in a variety of signaling pathways like cellular energy metabolism, aging, memory formation, circadian rhythm etc. (for review, see Johannessen et al., 2004). Using multiple isoforms of a protein to achieve functional diversity is a widely used strategy during the course of molecular evolution (for review, see Graveley, 2001; Touriol et al., 2003; Perrin and Ervasti, 2010). To test whether the above learning paradigm would allow us to parse out the isoform/s of CRH-1 required for LTM, we first tested the *creb1/crh-1-null* mutant, *crh-1(tz2)* (depicted in Fig. 2*A*). We observed that trained WT worms showed significantly reduced CI compared with *creb1/crh-1-null* mutants (Fig. 2*B*).

The *crh-1* gene encodes seven different isoforms (*crh-1 a-g*) (Wormbase gene: WBGene00000793). We were able to clone cDNA for six of the seven isoforms (*crh1 a-f*). We performed a quantitative PCR experiment to examine the expression levels of the different isoforms of CRH-1 during the early adult stage of *C. elegans*. We observed strikingly variable expression levels for the various isoforms indicating a possibility of functional diversity among the different isoforms (Fig. 2*C*). To further investigate the function of CREB1/CRH-1 in memory, different CRH-1 isoforms, i.e., *crh-1 (a-f*) were cloned under the pan-neuronal *rab-3* promoter and their ability to rescue the LTM defect in *creb1/crh-1-null* mutants was tested (Fig. 2*D*). Five of the six CRH-1 isoforms (CRH-1a-e) are full-length proteins having both the KID (kinase inducible domain) and the bZIP (DNA binding) domain, these five isoforms differ only in the N-terminal 30 aa, whereas CRH-1f is a truncated protein lacking the N-terminal KID motif (Fig. 1-1*D*). We observed rescue of LTM formation in case of CRH-1c and CRH-1e-expressing animals. Moreover, pan-neuronal expression of the repressor isoform CRH-1f in WT animals inhibited memory formation (Fig. 2*D*).

To negate the potential artifacts of spatiotemporal mis-expression and/or overexpression because of extrachromosomal arrays, we generated CRISPR mutants for the different *crh-1* isoforms and tested them for LTM defects. Deletion of a, b, and d isoforms by removing their first exon had no effect on LTM formation, whereas *C. elegans* without c and e isoforms were defective in LTM formation (Fig. 2*E*).

### Expression of CRH-1e in the RIM neurons rescues the learning defects of *creb1/crh-1* mutants

**Figure 3.**
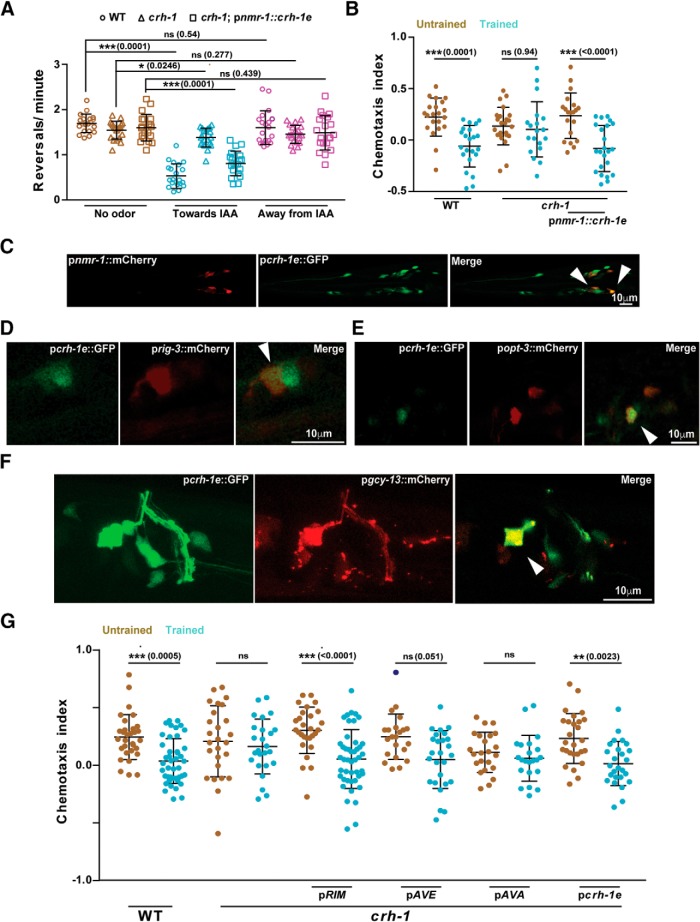
CRH-1e expression in the RIM or AVE interneurons is sufficient to rescue the associative learning phenotype seen in *crh-1* mutants. ***A***, Scatter plot showing reversal frequency of worms while they were moving either in odor free conditions, toward or away from the IAA point source. Each data point represents average reversal events per minute for individual animals counted over 10 min of chemotactic movement. ***B***, Chemotaxis indices for WT, *crh-1*, and p*nmr-1::crh-1e* rescue line. ***C***, Confocal microscope images showing overlapping expression of *crh-1e* promoter with neurons showing expression with the *nmr-1* promoter. Arrowheads indicate points of coexpression. ***D***–***F***, Confocal microscope images showing overlapping expression of *crh-1e* promoter with neuron-specific promoters; p*rig-3* (AVA), p*opt-3* (AVE), and p*gcy-13* (RIM). ***G***, Scatter plot showing chemotaxis indices of WT, *crh-1*, and rescue lines expressing CRH-1e under the promoters; *gcy-13* (RIM), *opt-3* (AVE), *rig-3* (AVA), and *crh-1e*. Error bars are SD. Multiple comparisons were done using one-way ANOVA and *p* values were adjusted using Bonferroni method. Data points represented with dark blue dots in the scatter plots represents outliers identified using Grubb's method (α = 0.05). *p < 0.05, **p < 0.01, ***p < 0.001, ns, *p* > 0.05.

Under stimulus-deficient environments, the movement of *C. elegans* can be described as a random walk. The presence of an attractant/repellent introduces a bias in this random walk strategy whereby the animal can suppress or enhance its reversal and turn frequency depending on whether it is moving toward or away from the attractant/repellent (Pierce-Shimomura et al., 1999). To test whether naive *creb1/crh-1-null* mutants showed any differences in their random walk pattern, we compared the reversal frequency of WT and *creb1/crh-1-null* mutants while they were moving on an NGM agar plate in response to an IAA gradient. Our results indicated that *creb1/crh-1-null* mutants were defective in modifying their reversal behavior in response to the varying attractant concentrations that they were experiencing while moving (Fig. 3*A*). Reversal behavior in *C. elegans* is controlled by the command interneurons (AVA, AVD, AVE) and the RIM interneurons (Chalfie et al., 1985; Gray et al., 2005; Piggott et al., 2011). To test whether CRH-1e is required in these neurons we tested a transgenic line expressing CRH-1e under the control of *nmr-1* promoter, which is expressed in five sets of neurons including AVA, AVD, AVE, and RIM (Brockie et al., 2001). This line could largely rescue the reversal defect as well as the memory deficits observed in the *creb1/crh-1-null* mutants (Fig. 3*A*,*B*).

The presence of a unique 5′ UTR on CRH-1e mRNA along with its low mRNA expression levels (Wormbase gene: WBGene00000793; Fig. 2*C*) suggests that this promoter sequence may be different from the other *crh-1* isoforms. We expressed GFP under the control of a 2.7 kb DNA sequence upstream of the CRH-1e translation start site (henceforth termed p*crh-1e*). Consistent with remarkably low levels of expression in qPCR measurements (Fig. 2*C*), we could observe the GFP expression in the p*crh-1e*::GFP line in only a few head neurons, expression in the head neurons was also seen previously by Kimura et al. (2002) who had done *in situ* hybridization of the *crh-1* gene. Colocalization experiments showed that the overlap in expression pattern of p*nmr-1* was largely restricted to three pairs of neurons (Fig. 3*C*). Based on the position of the overlapping neuron, we assessed that localization was seen in the AVA, AVE, and RIM interneurons. The same was confirmed by coexpression experiments using neuron-specific promoter marker lines for AVA (p*rig3*::mCherry), AVE (p*opt-3*::mCherry), and RIM (p*gcy-13*::mCherry; Fei et al., 2000; Ortiz et al., 2006; Feinberg et al., 2008). We found that p*crh-1e*::GFP localized with all these neurons (Fig. 3*D–F*). To test where CRH-1e is required for memory formation we expressed CRH-1e under the control of *crh-1e*, *rig-3*, *opt-3*, and *gcy-13* promoters. When tested for rescue of the associated memory phenotype with IAA and heat, we found that the *crh-1e*, the *gcy-13* and to a lesser extent the *opt-3* promoters could rescue the learning defects seen in *creb1/crh-1* mutants (Fig. 3*G*). Expressing CRH-1e in AVA using the *rig-3* promoter did not rescue the associative memory defects of *creb1/crh-1* mutants (Fig. 3*G*).

A recent study has shown that the CRH-1a isoform is broadly expressed along the *C. elegans* body and rescued the aging defects seen in *creb1/crh-1* mutants (Chen et al., 2016). We were interested in seeing whether CRH-1a was expressed in the command interneurons and in the RIM interneuron. To look for the localization of the *crh-1a* promoter in these neurons we made a p*crh-1a*::GFP construct and assessed for the colocalization between p*crh-1a*::GFP and p*nmr-1*::mCherry. Our results indicated that there was no overlap between p*crh-1a* expression and the command interneurons (data not shown) corroborating our hypothesis of functional diversity among CRH-1 isoforms.

### CRH-1 is required at the time of training

**Figure 4.**
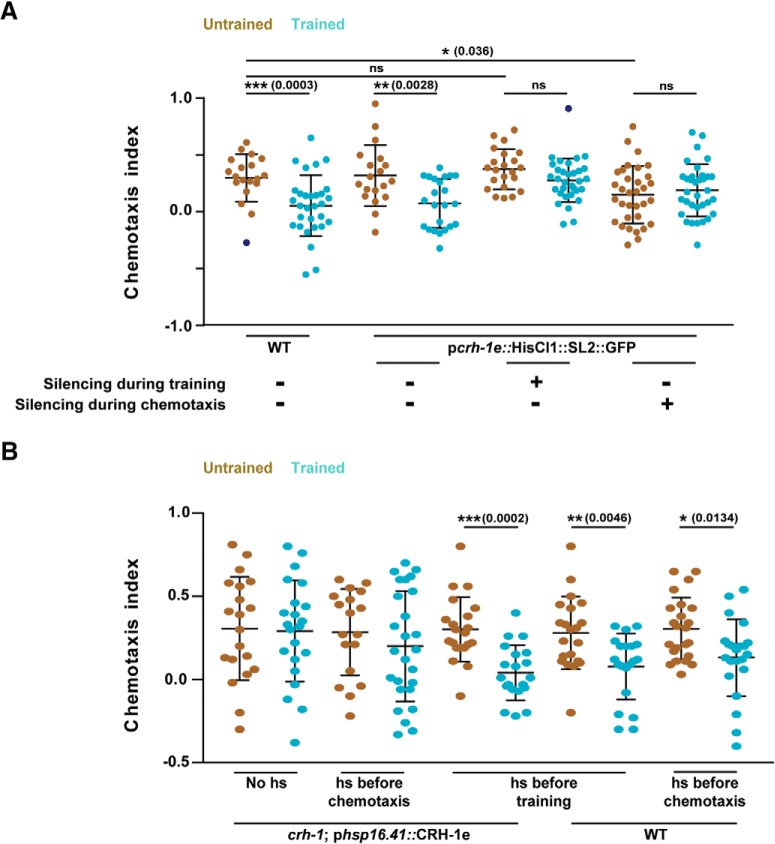
CRH-1e-expressing neurons are required for normal associative learning. ***A***, Scatter plot showing chemotaxis indices of *C. elegans* under the condition of histamine mediated neuronal silencing. ***B***, Chemotaxis indices of WT and *crh-1-null* mutants expressing CRH-1e under the control of the heat shock promoter *hsp-16.41*. Error bars are SD. Multiple comparisons were done using one-way ANOVA and *p* values were adjusted using Bonferroni method. Data points represented with dark blue dots in the scatter plots represents outliers identified using Grubb's method (α = 0.05). hs, Heat Shock. *p < 0.05, **p < 0.01, ***p < 0.001, ns, *p* > 0.05.

To study the temporal requirement of CRH-1e, we asked whether the activity of the CRH-1e-expressing neurons is required at the time of training. We expressed HisCl1 under the control of p*crh-1e* in WT animals and silenced the HisCl1-expressing neurons by growing the *C. elegans* on histamine-containing plates while executing the training protocol (from 10 min before training to 2 h after training) or during chemotaxis (20 h after training till the end of the assay). Memory formation was completely abolished in the animals with silenced neurons during training while silencing during chemotaxis resulted in defective chemotaxis even in naive animals (Fig. 4*A*).

To test the requirement of CRH-1e during the acquisition/consolidation phase of memory formation, we expressed CRH-1e under the control of the heat shock promoter (p*hsp16.41*) and used these animals to rescue the *creb1/crh-1* mutant phenotype. CRH-1e induction was done either 3 h before training or 3 h before chemotaxis. We observed that CRH-1e induction before training could largely rescue the learning defects, whereas induction before chemotaxis could not rescue the learning defect (Fig. 4*B*). These results suggest that CRH-1e is required at the time of acquisition and/or consolidation phase of memory formation in *creb1/crh-1* mutant animals.

### Lysine247 and lysine266 are key amino acid residues required for memory related functions of CRH-1

**Figure 5.**
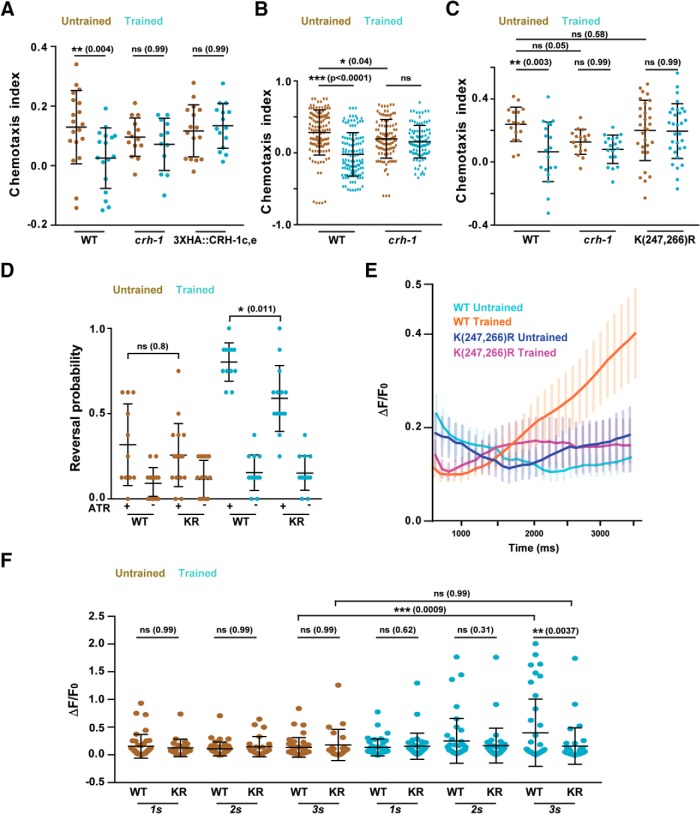
Lysine 247 and 266 residues are required for normal learning but not required for innate chemotaxis in *C. elegans*. ***A***, Chemotaxis indices of WT, *crh-1-null* mutants, and N-terminal 3xHA tagged. CRH-1c and CRH-1e. The tag was appended using CRISPR on N-terminal of CRH1c and CRH-1e translation start codon. ***B***, Scatter plot showing collated chemotaxis index values of WT and *crh-1-null C. elegans* used in this study. Data were pooled only form the experiments that used both strains. ***C***, Chemotaxis indices of WT, *crh1-null* mutants, and *crh-1*(K247R/K266R) mutants. ***D***, Reversal probability of *C. elegans* when AWC is excited with blue light. Blue light was illuminated for 3 s and reversal events were quantified. If the worm executed reverse movement for one body length, it was counted as a reversal event. Each *C. elegans* was illuminated with blue light 8 times with 20 s (***s***) intervals between subsequent stimulations. Each dot in the scatter plot indicates the reversal probability of a single animal under observation. ***E***, Plot showing Calcium traces of the AVA neuron in response to ChR2-mediated AWC activation. Mean values are plotted against time. Error bars are SEM. *n* = 25–41 for each genotype. ***F***, Scatter plot comparing AVA excitation in response to AWC activation at three time points. The figure represents a subset of data (1, 2, 3 s) from ***E***. Error bars are SD except in ***E*** (SEM). Multiple comparisons were done using one-way ANOVA and *p* values were adjusted using Bonferroni (***A***, ***D***, ***F***) and Dunnett's (***B***, ***C***) method. *p < 0.05, **p < 0.01, ***p < 0.001, ns, *p* > 0.05.

Most of the critical amino acid residues implicated in CREB1 activation are present in CRH-1a, b, and d isoforms. CRH-1c and e differ from CRH-1a, b, and d isoforms in having a much smaller and featureless N-terminal region. We hypothesized that the absence of these additional N-terminal residues is crucial for the functioning of CRH1c and CRH-1e in LTM related processes. We added an inert 3xHA tag to the N-terminal of CRH-1c and e (3xHA::CRH-1c,e) using CRISPR and tested these worms for the formation of LTM. Our experiments suggest defective memory formation in *C. elegans* with a modified N-terminal in CRH-1c and e (Fig. 5*A*).

Null mutants of *creb1/crh-1* are defective in LTM formation. Naive *creb1/crh-1 C. elegans* also show reduced chemotaxis indices compared with WT animals (Fig. 5*B*). This defect is partially because of the inability of *creb1/crh-1* mutant animals to modulate reversal frequency in response to chemical gradients (Fig. 3*A*). Therefore, observed learning defects in the *creb1/crh-1* mutant *C. elegans* appears to be the compounded effect of learning deficit as well as defective innate chemotactic behavior.

There are reports of dendritic localization of CREB1 (Crino et al., 1998). This increases the possibility of nuclear localization of CREB1 in response to dendritic stimulation. Moreover, SUMOylation-dependent nuclear localization of CREB1 has been found to be critical for LTM formation in mouse (Chen et al., 2014). K285 and K304 have been identified as CREB1 SUMO acceptor sites in mouse (Comerford et al., 2003). To test for the possible involvement of these residues we generated K247R/K266R (corresponding to K285 and K304 in mouse CREB1) *C. elegans* using CRISPR. These mutant animals were trained and tested for the LTM phenotype. We observed that K247R/K266R mutant worms were defective in LTM formation, whereas their naive behavior was comparable to that seen in WT animals (Fig. 5*C*). Because CRH-1c and e are the only isoforms that appear to be involved in the learning process in our experiments, it is conceivable that K247R/K266R mutations affect the function of these isoforms in the learning process.

The ability to modulate reversal frequency in response to chemical gradients is a primary factor in mediating chemotactic behaviors in worms. We tested the effect of K247R/K266R mutations on the ability of *C. elegans* to initiate reversals in response to AWC activity. We optogenetically activated the AWC neuron and recorded the reversal probability of WT and K247R/K266R animals under naive and trained conditions. Naive WT and K247R/K266R animals showed similar reversal frequencies while trained worms showed significantly increased reversal frequencies with the K247R/K266R animals showing lower reversal probability compared with WT control animals (Fig. 5*D*). To understand whether K247R/K266R mutations have any effect on AVA activity in response to AWC activity we optogenetically activated AWC for 3.0 s while simultaneously imaging AVA using GCaMP5G. While trained WT *C. elegans* showed significant increase in the probability of AVA firing upon AWC activation there was no such increase seen in case of K247R/K266R (Fig. 5*E*,*F*, KR) animals. These data suggest the involvement of K247 and K266 residues in regulating the AVA firing and reversal probability of worms under specific training conditions.

## Discussion

The role of CREB1 is well documented in the studies on learning and memory across phyla. It has been described as one of the main inducers of immediate early genes (IEGs) expressed in response to experience-dependent neural activation (for review, see Alberini, 1999). However, functional pleiotropy of CREB1 remains a major bottleneck in studying CREB1-dependent IEGs that are activated specifically in response to experience-dependent neural activation. Here, we describe the functional specialization of CRH-1 isoforms in *C. elegans*. Our experiments show that *C. elegans* lacking CRH-1c and CRH-1e are defective in LTM formation while having normal innate chemotaxis function. Null mutants of *creb1/crh-1* are defective in showing innate as well as learned behavior. Restricted expression of CRH-1e in a small subset of neurons indicates functional specialization of the isoform in *C. elegans*. In *Aplysia* and *Drosophila*, it has been shown that different isoforms of CREB1 can repress and facilitate the process of memory formation (Yin et al., 1995; Yin and Tully, 1996; Bartsch et al., 1998). However, what separates CRH-1 isoforms from their CREB1 orthologs is their remarkable sequence similarity yet striking functional diversity. Spatial segregation is an efficient way of exploiting different functional properties of proteins. CRH-1e and CRH-1a have different expression patterns (this work; Chen et al., 2016) and this spatial segregation can explain their functional segregation. However, this might not be the whole story here because pan-neuronal expression of CRH-1e cDNA could rescue the memory defects of *creb1/crh-1-null* mutants; while expressing CRH-1a, the isoform that could rescue aging defect of null animals (Chen et al., 2016), could not rescue memory defects. Further, a small featureless sequence like a 3xHA epitope tag that is routinely used in various biochemical and immunohistochemical assays without affecting the function of most proteins could disrupt the memory related functions of CRH-1c and CRH-1e. It is possible that these small differences result in significantly altered tertiary structures as seen in other proteins (Goda et al., 2000; Korepanova et al., 2001), hence affecting activation dynamics or subcellular localization of the different protein isoforms (Crino et al., 1998). It appears that the absence of these additional N-terminal amino acids from CRH-1a, b, and d enables the CRH-1c and e isoforms to interact with new binding partners either by allowing CRH-1c and e a different tertiary structure or by removing steric hindrance. CRISPR mutants with K247R/K266R are defective in learned responses while having normal innate response to IAA gradient. SUMOylation of homologous residues has been shown to be associated with LTM defects in rats undergoing water maze test. It is yet to be seen whether K247 and K266 undergo SUMOylation in *C. elegans*.

Lau et al. have shown that the NMDA-type glutamate receptor-dependent associative memory defects seen is *nmr-1* mutants is rescued by expressing NMR-1 in the RIM interneuron among other neurons (Lau et al., 2013). Previous work has shown that the glutamate receptor, GLR-1 function is necessary for LTM formation in *C. elegans* (Rose et al., 2003). AVE and RIM both express NMR-1 and GLR-1 and are connected to each other through gap junctions (Brockie et al., 2001; Kano et al., 2008; Kawano et al., 2011; Piggott et al., 2011; Lau et al., 2013). This along with our data makes it conceivable that RIM and AVE could be functioning together in the process of memory formation. Further, Jin et al. (2016) have recently shown that the interneurons RIM and AIB are required for CREB/CRH-1-mediated imprinted memory formation, whereas the interneurons AIY and RIA are required for retrieval of the memory. Our results also show that presence of functional CRH-1e in RIM or AVE is sufficient for overcoming LTM formation defect in *creb1/crh-1-null* worms. Both long-term associative memory and long-term habituation implicate CREB1/CRH-1 in RIM neurons (Timbers and Rankin, 2011). Even though different neural circuits are activated by the training paradigms used in these studies, both paradigms operate in part by modulating the naive reversal behavior in response to a cue. Consistent with these observations our experiments implicate K247 and K266 in modulating reversal probability in trained worms upon AWC activation through channel rhodopsin. Moreover, CRH-1 (K247R/K266R) trained animals have diminished AWC-dependent AVA activation as measured by GCaMP5-mediated calcium imaging. This suggests that CRH-1-dependent genes might provide substrates for experience-dependent modulation of reversal behaviors in *C. elegans*.

Channelrhodopsin-mediated AWC activation highlights the importance of K247R/K266R residues in the modulation of reversal probability/AVA excitability exclusively in an experience-dependent manner. Under these experimental conditions, these mutations express themselves exclusively when worms are subjected to the training protocol. However, these results may not be true representative of natural phenomena operating during chemotactic movement of worms. Physiologically AWC are OFF type neurons, they are activated in response to step down of attractant concentration (Troemel et al., 1999). This makes the results of our optogenetics experiments counterintuitive. Our optogenetics experiment suffers from two technical problems; the promoter used for expression of ChR2, p*str-2*, only expresses in one AWC neuron, AWC^ON^ (Troemel et al., 1999). Hence, we could only activate one AWC neuron, which is unnatural for worms sensing IAA. Second is our inability to localize ChR2 to the subcellular position of chemosensors. The receptor for IAA is unknown. However, we know that ODR-10, a GPCR for diacetyl, is primarily localized to the ciliated endings of AWA neurons (Sengupta et al., 1996). We can assume similar localization patterns for IAA receptors. Sensory neurons in worms also receive information from other neurons hence technically also act as interneurons and due to graded potential, they process and transmit this information in a complex way. Because of localization of channelrhodopsin throughout the surface of the neuron the output of optogenetically activated neuron lacks any resemblance to the output of ligand-activated neuron. However, we do observe reversal probability of optogenetically activated naive worms that are consistent with the previous studies making the results that we got from trained group of animals difficult to explain in terms of experimental artifacts alone (Cho et al., 2016). These problems could be overcome by using a more natural method using ligand-based neuron activation in microfluidics chambers.

AVA activity is highly correlated with reversal behavior in *C. elegans* (Gray et al., 2005; Ben Arous et al., 2010; Bhardwaj et al., 2018). The ability to modulate AVA activity in response to sensory neuron activation provides for an important control point of behavioral modification through reversal modulation. In a parallel set of experiments, we have found that learning in worms is specific to the cues that are presented at the time of training. Therefore, given the strong correlation of AVA activity and reversal event it was surprising to find that expression of CRH-1e RIM and AVE was sufficient to restore LTM defect in *creb1/crh-1-null* mutants. AVA is heavily connected with AVE (44 incoming chemical synapses) and RIM (6 electrical synapses, 3 incoming chemical synapses; White et al., 1986). Any change in RIM/AVE due to activity-dependent CRH-1 activation in response to different sensory cues is likely to produce similar effects on AVA neurons. There are two probable explanations. One, the rescue of LTM defect that we observed in our experiments is an artifact of the experimental methods used. Because specificity of promoters is largely determined by visible fluorescent protein reporter gene expression in a particular cell, it is likely that subvisible expression at other sites is responsible for the LTM defect rescue. Second, learning but not its specificity is a function of CRH-1. If this is true, then it is likely that by studying CRH-1-dependent IEGs in *C. elegans* we will only be able to study the somewhat mechanical aspect of memory manifesting at the terminal end of neural hierarchy just before behavior execution.
