# Supplementary figures and images for: Differential Regulation of Innate and Learned Behavior by Creb1/Crh-1 in Caenorhabditis elegans
Source: J Neurosci. 2019 Oct 2;39(40):7934–46. doi: 10.1523/JNEUROSCI.0006-19.2019 (PMC6774408; doi:10.1523/JNEUROSCI.0006-19.2019)

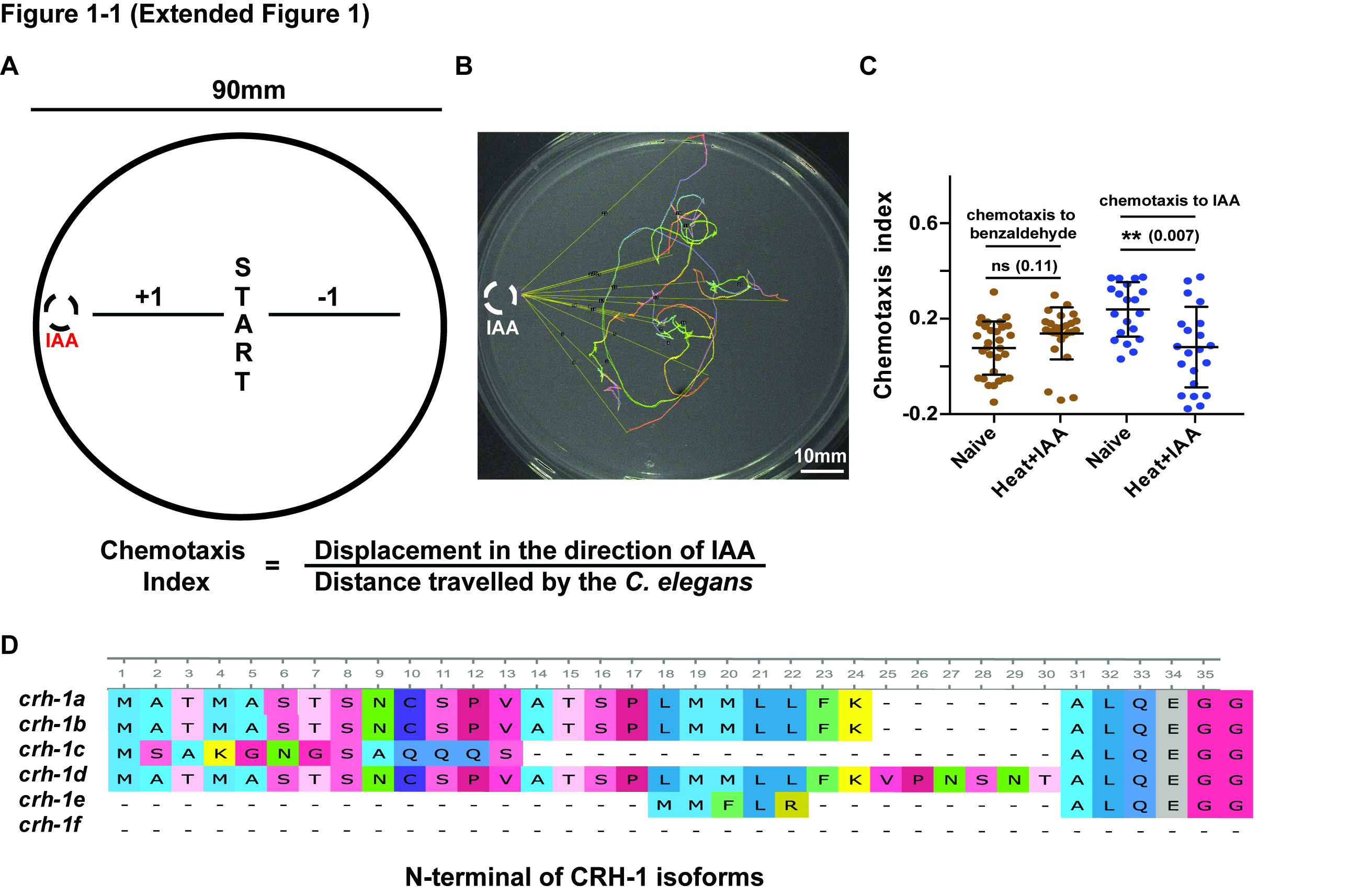

Supplement: Figure 1-1 [file zns999191947so1.tif]
